# Spatial Spillover Effects of Resource Misallocation on the Green Total Factor Productivity in Chinese Agriculture

**DOI:** 10.3390/ijerph192315718

**Published:** 2022-11-25

**Authors:** Jiangfeng Hu, Xiaofang Zhang, Tingting Wang

**Affiliations:** 1 Chongqing Academy of Social Sciences, Chongqing 400020, China; 2 Pass College, Chongqing Technology and Business University, Chongqing 401520, China; 3 Economic Law School, Southwest University of Political Science and Law, Chongqing 401120, China

**Keywords:** agricultural economics, resource misallocation, green total factor productivity, sequential DEA, spatial panel model

## Abstract

Continuous resource misallocation not only results in total factor productivity loss but also leads to ecological degradation. Therefore, in the process of changing from extensive growth to intensive growth, Chinese agriculture should pay attention to the problem of resource misallocation. There is currently a lack of relevant research, especially concerning the spatial spillover effects of resource misallocation at the city level. To fill this gap, we employ a spatial panel model for empirical testing on the basis of measuring agricultural green total factor productivity (GTFP) in 306 cities in China from 1996–2017. We found that there is positive spatial autocorrelation in Chinese agricultural GTFP, but it decreases year by year. Misallocation in land, labor, machinery and fertilizer all directly hinder the local GTFP. The eastern is mainly negatively affected by neighbor resource misallocation, while the central and western are mainly negatively affected by local resource misallocation. Finally, the indirect effect of neighbor resource misallocation on GTFP gradually shifts from inhibiting effect to a facilitating effect with increasing spatial distance. These findings have clear policy implications: Chinese government should strengthen agricultural green technology innovation and diffusion, strengthen environmental regulation and promote the free movement of labor between regions and sectors.

## 1. Introduction

Since its reform and opening up (reform and opening up is a policy of domestic reform and opening up that China began to implement at the Third Plenary Session of the Eleventh Central Committee in December 1978), China has made great achievements in agriculture, feeding 1/5 of the world’s population on less than 10% arable land [1]. It even achieved what was described as “twelve consecutive increases” in food production during the period 2003–2015, strongly responding to the question of “who feeds China”. However, while Chinese agriculture is growing rapidly, it is also facing many serious problems, such as excessive consumption of resources, serious non-point source pollution and the agroecological deterioration [2,3]. In order to reverse the unsustainable situation of agricultural development, the Chinese government has introduced a series of laws, regulations and policies to promote green growth (namely, to make resources efficient, clean and recyclable without slowing down the growth rate [4]) in agriculture. The promulgation of these policies has undoubtedly provided useful ideas and feasible solutions to the dilemma of food security and ecological safety faced by China. It is foreseeable that green growth will become an inevitable trend in Chinese agricultural development in the future.

However, in the process of changing from extensive growth to intensive growth, Chinese agriculture has had to pay attention to the problem of resource misallocation. It is generally accepted that in a perfectly competitive economy, homogeneous factors should have the same marginal returns, otherwise there will be a flow from the side with lower returns to the side with higher returns, and eliminating such gaps will eventually lead to achieving market equilibrium. If factor markets are distorted, the free flow of factors will be restricted, resulting in an inability to be allocated to where they are most efficient [5,6]. In this case, the economy would fail to achieve the Pareto optimal allocation and result in social efficiency loss [7,8]. According to Restuccia and Rogerson [9], Ouyang et al. [10] and Su and Liang [11], market segmentation, government regulation and lagging factor market reforms can all lead to resource misallocation. Especially in a government-dominated economy, lower factor prices help drive economic growth through increased factor inputs, so distorting factor markets are often used by governments as a policy tool for catch-up development strategies [6,12].

The Chinese agricultural sector has been largely dominated by the government in terms of resource allocation, with the flow of resources being regulated, and thus being unable to be allocated to the most efficient side (region or sector). This has led to an imbalance between marginal factor returns and factor prices, resulting in serious distortions in the allocation of agricultural production factors. Adamopoulos et al. [13] found that under the household contract responsibility system (HRS), rural land is allocated equally, ignoring differences in productivity in agriculture, making the degree of land resource misallocation in rural China worse over time, resulting in a 36–84% loss in additive TFP. Zhao [14] point out that the Rural Land Contract Law (RLCL) enacted by the Chinese government in 2003 prohibits land reallocation, which, while alleviating labor misallocation, also reduced farmers’ willingness to outsource their land, thereby exacerbating land misallocation and contributing to a 6% loss in total output.

The continuous resource misallocation not only results in TFP loss, but also leads to ecological degradation. On the one hand, lower factor price reduces producers’ willingness to improve the efficiency of resource use through technological innovation, leading to a stagnation of resource-saving technological progress [15,16]. On the other hand, a distorted price system fails to reflect the scarcity and opportunity of the cost of resources, and changes in factors of production, in turn, hinder the role of factor markets in optimizing resource allocation [17]. Based on their respective studies, scholars have found that resource misallocation or factor market distortions can aggravate pollution emissions [6,18] and haze pollution [19], reduce environmental efficiency [20], green total factor productivity [21], green technological progress [22] and energy efficiency [23]. For instance, Bian et al. [18] studied the impact of market segmentation on environmental pollution from the perspective of resource misallocation and found that market segmentation has significantly aggravated the misallocation of labor and capital resources, which led to environmental pollution. However, the existing literature is mainly based on provincial or industry-level data, and the spatial spillover effects of resource misallocation affecting agricultural GTFP have not been studied at the city level. To fill this gap, this paper empirically examines the impact of resource misallocation on green total factor productivity in Chinese agriculture using a spatial panel model based on balanced panel data from 306 cities in China from 1996–2017. It is helpful to provide theoretical support for solving the dilemma of food security and ecological safety in China from a resource allocation perspective and also have implications for developing countries with similar resource endowments and facing similar problems.

The paper contributes to the literature in three major ways. Firstly, this paper uses data from 306 cities in China as the research sample. Most of the existing studies on resource misallocation and total factor productivity use provincial and farmer-level data, and there is a lack of empirical studies at the city level. The motivation for using city-level data in this paper stems from two points: On the one hand, it is difficult to precisely capture the spatial spillover effects of resource allocation on the impact of agricultural GTFP due to the huge differences in cropping structure, economic development, policies and resource endowments among provinces. In contrast, there is relatively little variation in various aspects across cities in the same province, thus allowing the interference of external factors in the empirical results to be eliminated. On the other hand, data at the household level are limited by the method of sample collection and the perceptions of the respondents, which may lead to problems, such as sample selection bias. The data at the city level are directly sourced from official statistics, which can effectively avoid biased results caused by data distortion. For this reason, this paper collects a total of 306 cities in China from 1996–2017 as a balanced panel data for the study sample.

Secondly, this paper focuses its research on green growth in agriculture. Existing studies on the impact of resource misallocation on green growth have mainly focused on regions and industries [16,20,21,23], and the causal relationship between resource misallocation and green growth in the Chinese agricultural sector has not been studied. As the ballast and propeller of economic growth, social progress and national development, the green growth of agriculture determines the bottom line and potential of the country’s livelihood. In this paper, we refer to Chen et al. [22], Han et al. [24], Han et al. [25], Liu and Feng [26], Tang et al. [27], Zou et al. [28], Qu et al. [29] to account for agricultural non-point source pollution (unexpected output), and then measure agricultural GTFP using a sequential data envelopment analysis (DEA) method to provide indicator guarantees for subsequent empirical studies.

Thirdly, this paper uses a spatial panel model to examine the spatial spillover effects of resource misallocation on the impact of GTFP in agriculture. Existing studies have only considered resource misallocation to local regions or aggregate total factor productivity loss and have not yet focused on the spatial spillover effects of agricultural resource misallocation. Hao et al. [21] used a spatial panel model to find that resource (labor and capital) misallocation has a negative impact on GTFP in 30 Chinese provinces. However, the study was not on agriculture, and the marginal effects of resource misallocation were not decomposed into direct and indirect effects, making it impossible to distinguish the spillover effects of resource misallocation on agricultural GTFP as coming from the local region or from other regions. Based on this, this paper uses a spatial panel model to empirically test the spatial spillover effect of resource misallocation on the impact of agricultural GTFP, and decomposes the regression coefficients into total, direct and indirect effects. The direct effect represents the net effect of changes in the independent variables in the local region, the indirect effect represents the effect of changes in the independent variables in other regions on the local region, and the total effect is the sum of the direct and indirect effects.

The remainder of the paper is organized as follows. Section 2 describes the methods, variables and data. Section 3 presents panel data test results. Section 4 presents and discusses the empirical results, and Section 5 ends with conclusions.

## 2. Materials and Methods

### 2.1. Methods

#### 2.1.1. Sequential DEA

The concept of “green total factor productivity” was first proposed by the United Nations Environment Programme [30], which refers to the comprehensive utilization efficiency of all input factors in the social production process, including “factor utilization efficiency” and “environmental efficiency”. The former represents the output level brought by factors input, while the latter represents that the matching combination of each factor can meet the bearing range of the ecological environment, emphasizing the coordinated development of economic growth and ecological environment. Green total factor productivity directly connects environmental impact and economic development and is an effective tool for the comprehensive evaluation of green production. Compared with total factor productivity, green total factor productivity is the efficiency level after deducting the negative impact on the environment, which can better reflect the real productivity level.

The DEA method is widely used to calculate GTFP because it does not require a specific production function and can include multiple inputs and outputs (expected and unexpected outputs). This paper uses each city as a decision module (DMU) to construct the production frontier. Assuming each DMU uses K inputs, $x_{\mathrm{kj}}\left(n=1,\;\ldots ,\;K\right)\;\epsilon {\;R}^{+}$. Where i represents the i-th city that has obtained M non-negative expected outputs, $y_{\mathrm{mj}}\left(m=1,\;\ldots ,\;M\right)\;\epsilon {\;R}^{+}$, U non-negative unexpected outputs, and $b_{\mathrm{uj}}\left(u=1,\;\ldots ,\;U\right)\;\epsilon {\;R}^{+}$. At the same time, in order to eliminate the degradation of the pseudo technology of the traditional DEA method, the current output set has nothing to do with the previous feasible technology; this paper uses the method of Shestalova [31] and adopts the output-oriented sequential DEA to construct the technological frontier.

(1) $${\bar{P}}^{t}\left(x\right)=\left\{\left(y,\;b\right):\;y\leq {\bar{Y}}^{t}\left(1+\beta^{t}\right),\;b={\bar{B}}^{t}\left(1-\beta^{t}\right),\;x\geq {\bar{X}}^{t}\lambda ,\lambda \geq 0\right\}$$

where λ is the weight, ${\bar{X}}^{t}=\left(\;X^{t_{0}},\cdots ,X^{t-1},X^{t}\right)=\left({\bar{X}}^{t-1},X^{t}\right),\;{\bar{Y}}^{t}=\left(\;Y^{t_{0}},\cdots ,Y^{t-1},Y^{t}\right)=\left({\bar{Y}}^{t-1},Y^{t}\right),\;{\bar{B}}^{t}=\left(\;B^{t_{0}},\cdots ,B^{t-1},B^{t}\right)=\left({\bar{B}}^{t-1},B^{t}\right)$, and $t_{0}$ is the first period, for which observations on inputs and outputs are available. Therefore, the linear program that defines the distance function relative to the sequential frontier becomes:

(2) $$\begin{array}{c} {\vec{D}}_{o}^{t}\left(x^{t},y^{t},b^{t};y^{t},-b^{t}\right)=max\beta^{t}\\ s.t.\;\overset{t}{\underset{p=1}{\sum }}\overset{N}{\underset{i=1,}{\sum }}\lambda_{i}^{p}X_{ki}^{p}\leq x_{ki}^{t},k=N,L,M,F\\ \overset{t}{\underset{p=1}{\sum }}\overset{N}{\underset{i=1,}{\sum }}\lambda_{i}^{p}Y_{mi}^{p}\geq \left(1+\beta^{t}\right)\cdot y_{mi}^{t}\\ \overset{t}{\underset{p=1}{\sum }}\overset{N}{\underset{i=1}{\sum }}\lambda_{i}^{p}B_{ui}^{p}=\left(1-\beta^{t}\right)\cdot b_{ui}^{t}\\ \lambda_{i}^{p},\beta^{t}\geq 0,\;for\;all\;i,\;k;\;i=1,\cdots ,N \end{array}$$

In this paper, GTFP is further decomposed by Malmquist–Luenberger index:

(3) $${\mathrm{GTFP}}_{t}^{t+1}={\left\{\frac{1+{\vec{D}}_{o}^{t}{(x}^{t}{,y}^{t},b^{t};g^{t})}{1+{\vec{D}}_{o}^{t}{(x}^{t+1}{,y}^{t+1},b^{t+1};g^{t+1})}\times \frac{1+{\vec{D}}_{o}^{t+1}{(x}^{t}{,y}^{t},b^{t};g^{t})}{1+{\vec{D}}_{o}^{t+1}{(x}^{t+1}{,y}^{t+1},b^{t+1};g^{t+1})}\right\}}^{\frac{1}{2}}$$

The GTFP is further decomposed into an efficiency change index (EFFCH) and a technology change index (TECH).

(4) $${\mathrm{EFFCH}}_{t}^{t+1}=\frac{1+D_{o}^{t}{(x}^{t}{,y}^{t},b^{t};g^{t})}{1+D_{o}^{t+1}{(x}^{t+1}{,y}^{t+1},b^{t+1};g^{t+1})}$$

(5) $${\mathrm{TECH}}_{t}^{t+1}={\left\{\frac{1+D_{o}^{t+1}{(x}^{t}{,y}^{t},b^{t};g^{t})}{1+D_{o}^{t}{(x}^{t}{,y}^{t},b^{t};g^{t})}\times \frac{1+D_{o}^{t+1}{(x}^{t+1}{,y}^{t+1},b^{t+1};g^{t+1})}{1+D_{o}^{t}{(x}^{t+1}{,y}^{t+1},b^{t+1};g^{t+1})}\right\}}^{\frac{1}{2}}$$

(6) $${\mathrm{GTFP}}_{t}^{t+1}={\mathrm{EFFCH}}_{t}^{t+1}\times {\mathrm{TECH}}_{t}^{t+1}$$

GTFP, EFFCH and TECH greater than (less than) 1 represent GTFP growth (decrease), technical efficiency is improved (decreased), and if technological progress is equal to 1, it means that the period from t to t+1 is unchanged. It should be noted that TECH is greater than or equal to 1, except that the base period TECH may be less than 1.

#### 2.1.2. Spatial Panel Model

In order to verify the impact of resource misallocation on agricultural GTFP, this paper constructs the following benchmark model:

(7) $${\mathrm{GTFP}}_{\mathrm{it}}=\alpha +\beta \cdot {\mathrm{LnMis}}_{\mathrm{it}}+\gamma \cdot {\mathrm{Control}}_{\mathrm{it}}+\varepsilon_{\mathrm{it}}$$

where LnMis=[LnMis_L,LnMis_N,LnMis_M,LnMis_F] denotes the matrix of resource misallocation variables, Control is the control variable and $\varepsilon_{\mathrm{it}}$ is the residual term; i denotes the i-th city, t denotes time and Ln denotes taking the natural logarithm.

Considering the resource misallocation worthy of this paper, it means that the spatial flow of resources is restricted from achieving optimal allocation. Thus, resource misallocation is spatially correlated. Therefore, we refer to Elhorst [32] and use a spatial econometric model, which is undoubtedly more in line with reality. In addition, the resource misallocation variable contains a dependent variable component that is inversely influenced by total factor productivity, i.e., there is endogeneity. In this paper, the previous period of GTFP is used as the dependent variable.

(8) $${\mathrm{GTFP}}_{\mathrm{it}+1}=\alpha +\beta \cdot {\mathrm{LnMis}}_{\mathrm{it}}+\gamma \cdot {\mathrm{Control}}_{\mathrm{it}}+\mu_{\mathrm{it}}+\lambda \cdot W\cdot Y_{\mathrm{it}}$$

where $W=W_{\mathrm{ij}}=\{\begin{array}{c} 1,\;i\neq j\\ 0,\;i=j \end{array}$ is the spatial adjacency matrix. If the i-th municipality has the same boundary as the j-th municipality, then $W_{\mathrm{ij}}=1$, otherwise $W_{\mathrm{ij}}=0$; $\mu_{\mathrm{it}}=\rho \cdot W\cdot \mu_{\mathrm{it}}+\varepsilon_{\mathrm{it}}$ is the spatial error term and $\varepsilon_{\mathrm{it}}$ is the random perturbation term. If λ=0 and ρ≠0, then it shows the spatial error model (SEM); if λ=0 and ρ≠0, then it shows the spatial lag model (SAR); if λ≠0 and ρ=0, then it shows the more general spatial lag and error model (SAREM).

Finally, considering that the degree of resource misallocation in the neighbor regions may affect the level of green growth in the region, this paper further adds the spatial lag term of the independent variables on the basis of Equation (8) to obtain the spatial Durbin model (SDM).

(9) $${\mathrm{GTFP}}_{\mathrm{it}+1}=\alpha +\beta \cdot {\mathrm{LnMis}}_{\mathrm{it}}+\gamma \cdot {\mathrm{Control}}_{\mathrm{it}}+\theta_{1}\cdot W\cdot {\mathrm{LnMis}}_{\mathrm{it}}+\theta_{2}\cdot W\cdot {\mathrm{Control}}_{\mathrm{it}}+\lambda \cdot W\cdot Y_{\mathrm{it}}+\mu_{\mathrm{it}}$$

It should be noted that the use of OLS to estimate the spatial model will lead to bias and inefficiency. In order to avoid these problems, we refer to Lee [33] and use maximum likelihood (ML) to estimate the model.

#### 2.1.3. Resource Misallocation

According to the theoretical model of Hsieh and Klenow [34] and others, there is a misallocation of resources due to the presence of distortion $\tau_{\mathrm{it}}^{X}$. In this paper, the resource misallocation is defined as:

(10) $${\mathrm{Mis}}_{\mathrm{Xt}}=\frac{1}{1+\tau_{\mathrm{it}}^{X}},\;X=N,\;L,\;M,\;F$$

${\mathrm{Mis}}_{\mathrm{Xt}}=1$ when $\tau_{\mathrm{it}}^{X}=0$, indicating that there is no resource misallocation in the i-th city; otherwise, there is a misallocation. However, $\tau_{\mathrm{it}}^{X}$ is not directly observable in the reality. Hsieh and Klenow [34] argue that in a perfectly competitive market, the marginal return of the same factor should be equal for each individual, i.e., ${\mathrm{MVPX}}_{\mathrm{it}}={\mathrm{MVPX}}_{\mathrm{jt}}$. Ultimately, ${\mathrm{MVPX}}_{\mathrm{it}}={\mathrm{MVPX}}_{\mathrm{st}}$ where ${\mathrm{MVPX}}_{\mathrm{st}}$ is the factor marginal return in the s-th province. Therefore, the relative degree of misallocation of each factor can be defined as:

(11) $${\mathrm{Mis}}_{X}=\frac{{\mathrm{MVPX}}_{\mathrm{st}}}{{\mathrm{MVPX}}_{\mathrm{it}}}$$

(12) $$Y_{\mathrm{it}}=A_{\mathrm{it}}N_{\mathrm{it}}^{\alpha }L_{\mathrm{it}}^{\beta }M_{\mathrm{it}}^{\gamma }F_{\mathrm{it}}^{\varphi }$$

(13) $${\mathrm{MVPX}}_{\mathrm{it}}=\alpha \frac{P_{\mathrm{it}}Y_{\mathrm{it}}}{X_{\mathrm{it}}}$$

Similarly,

(14) $${\mathrm{MVPX}}_{\mathrm{st}}=\alpha \frac{P_{\mathrm{st}}Y_{\mathrm{st}}}{X_{\mathrm{st}}}$$

Substituting Equations (13) and (14) into Equation (11), respectively, we can find:

(15) $${\mathrm{Mis}}_{\mathrm{Xt}}=\frac{X_{\mathrm{it}}}{\sum_{i}X_{\mathrm{it}}}\frac{\sum_{i}P_{\mathrm{it}}Y_{\mathrm{it}}}{P_{\mathrm{it}}Y_{\mathrm{it}}}=\frac{X_{\mathrm{it}}}{X_{\mathrm{st}}}\frac{P_{\mathrm{st}}Y_{\mathrm{st}}}{P_{\mathrm{it}}Y_{\mathrm{it}}}$$

This paper assumes a high degree of substitutability of output between different cities within the same province. Thus, the output at the provincial level can be obtained by summing the output of cities.

(16) $$Y_{\mathrm{st}}=\underset{i}{\sum }Y_{\mathrm{it}}$$

Thus, $\frac{P_{\mathrm{st}}}{P_{\mathrm{it}}}=1$; substituting Equation (15), we can find:

(17) $${\mathrm{Mis}}_{\mathrm{Xt}}=\frac{X_{\mathrm{it}}}{X_{\mathrm{st}}}\frac{Y_{\mathrm{st}}}{Y_{\mathrm{it}}}$$

When ${\mathrm{Mis}}_{\mathrm{Xt}}=1$, it means that there is no misallocation, while ${\mathrm{Mis}}_{\mathrm{Xt}}>1$ or ${\mathrm{Mis}}_{\mathrm{Xt}}<1$ means that there is a misallocation. This indicates that the larger the absolute value of the difference between ${\mathrm{Mis}}_{\mathrm{Xt}}$ and 1, the greater the degree of resource misallocation. Therefore, this paper uses $\left|{\mathrm{Mis}}_{\mathrm{Xt}}-1\right|$ to indicate the degree of resource misallocation. Finally, we get Mis=[Mis_L,Mis_N,Mis_M,Mis_F]. When Mis=0, there is no resource misallocation; when Mis>0, there is a misallocation, and the greater the LnMis, the more serious the misallocation.

### 2.2. Materials

#### 2.2.1. Variable and Definition

##### Input and Output Variables

Measuring GTFP requires the use of expected output, unexpected output and input variables, and the selection of relevant variables in this paper follows the existing literature.

Agricultural output. Many scholars divide output into expected output (Y) and unexpected output (B). Expected output (Y): in this paper, total agricultural output is used to measure output and deflated using the 1978 price index of primary industry output. Unexpected output (B) mainly includes total phosphorus (TP), total nitrogen (TN) and chemical oxygen demand (COD). This paper refers to Chen et al. [22], Han et al. [24], Han et al. [25], Liu and Feng [26], Tang et al. [27], Zou et al. [28] and Qu et al. [29] for the unit survey assessment method using inventory analysis for accounting.

(18) $$\begin{array}{c} E_{j}=\underset{i}{\sum }{\mathrm{EU}}_{\mathrm{ij}}\times \eta_{\mathrm{ij}}\left({\mathrm{EU}}_{\mathrm{ij}},S\right)\\ =\underset{i}{\sum }{\mathrm{PE}}_{\mathrm{ij}}\times \rho_{\mathrm{ij}}\times \eta_{\mathrm{ij}}\left({\mathrm{EU}}_{\mathrm{ij}},S\right) \end{array}$$

where E is agricultural pollution emissions, ${\mathrm{EU}}_{i}$ is the indicator statistic for unit i (i=agricultural waste and fertilizer loss pollutants) pollutant j (j=TP, TN and COD), $\eta_{\mathrm{ij}}$ is the nutrient loss rate for unit i pollutant j, ${\mathrm{PE}}_{\mathrm{ij}}$ is the amount of agricultural pollution produced and $\rho_{\mathrm{ij}}$ is the pollution production coefficient, mainly determined by the unit and spatial characteristics that S determines.

**Figure 1:**
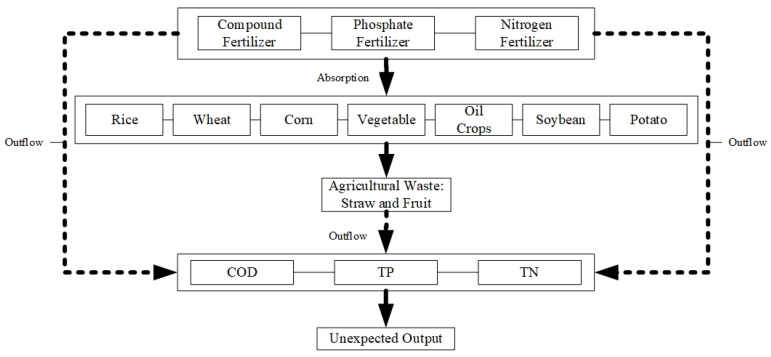
Unexpected output production system.

Figure 1 depicts the generation process of agricultural non-point source pollution. From 1978 to 2016, China’s total fertilizer-using increased by nearly six times [35], accounting for more than a 1/3 usage of global fertilizer, but the utilization rate was less than half of the world average [36], and long-term excessive use of agrochemicals will not be absorbed by plants to increase production but will lead to environmental pollution due to the loss of fertilizer [36,37]. In addition, due to small-scale decentralized agricultural operation, straw and waste fruits are difficult to be comprehensively utilized and will also cause environmental pollution if discarded.

For the pollution production coefficient of chemical fertilizer ($\rho_{\mathrm{ij}}$), this paper refers to the work Chen et al. [38] in which the calculations are performed according to the chemical composition of fertilizer conversion: The TN pollution production coefficients of nitrogen fertilizer, phosphorus fertilizer and compound fertilizer (the nutrient ratio of nitrogen, phosphorus and potassium is 1:1:1) are 1, 0 and 0.33, respectively. The TP pollution production coefficients of nitrogen fertilizer, potassium fertilizer and compound fertilizer are 0, 0.44 and 0.15, respectively. For the loss rate of chemical fertilizer ($\eta_{\mathrm{ij}}$), this paper refers to Chen et al. [38] to determine the results (see Table S1 in Supplementary Materials).

In order to ensure the integrity of the indicators and the quality of data, we refer to Chen et al. [22], Han et al. [24], Han et al. [25], Liu and Feng [26], Tang et al. [27], Zou et al. [28] and Qu et al. [29] who mainly consider straw produced by rice, wheat, corn, oil crops, soybeans and potatoes and also the solid waste produced by vegetables. Meanwhile, this paper refers to Chen et al. [38] to determine the pollution production coefficient (see Table S2 in Supplementary Materials) and nutrient loss coefficient (see Table S3 in Supplementary Materials) of different crops.

Agricultural input. Labor (N): In this paper, the number of people employed in agriculture is used as labor input, but as the available statistics do not distinguish the data on agricultural employees from those employed in agriculture, forestry, and animal husbandry and fishery, the total agricultural output value as a proportion of the total agricultural output value of agriculture, forestry, and animal husbandry and fishery is used as the weight in this paper to separate out the number of people employed in agriculture. Land (L): In order to reflect the situation of replanting and replacing crops, this paper uses the total sown area of crops. Machinery (M): In this paper, the total power of agricultural machinery is used. Fertilizer (F): This paper expresses fertilizer input as the discounted amount of fertilizer applied to agricultural production each year, including nitrogen fertilizer, phosphate fertilizer, potash fertilizer and compound fertilizer.

#### 2.2.2. Control Variables

The control variables in this paper include: rural population proportion (RPP), per capita GDP (PGDP), population density (PD), environmental regulation (ER), technological innovation (Patent) and foreign direct investment (FDI).

Rural population proportion (RPP): On the one hand, the shift of the rural population will ease the tense human–land relationship in the countryside and contribute to the increase in total factor productivity in agriculture. On the other hand, the rural population shift is only a result of China’s urbanization, and the encroachment of urban construction on agricultural land will lead farmers to applying large amounts of chemical fertilizer in order to increase food production on limited land, thus increasing agricultural surface pollution [37]. Based on this, this paper uses the ratio of the rural population to the total population at the municipal level as the rural population proportion (RPP) indicator. Per capita GDP (PGDP): Existing studies generally use per capita GDP to express the level of regional economic development and argue that higher levels of economic development facilitate access to agricultural production factors and advanced agricultural technologies [37,39]. The population density (PD): Overpopulation in cities will cause problems, such as over-consumption of resources, traffic congestion and occupation of arable land, which will affect the ecological environment. In this paper, the number of people per square kilometer is expressed. Environmental regulation (ER): Environmental regulation will crowd out normal investment, and thus be detrimental to the competitiveness of a country’s industries. On the other hand, environmental regulation will stimulate technological innovation, and the resulting technological innovation will contribute to green total factor productivity. This paper is expressed in terms of the total number of environmental protection establishments at the end of the year. Technological innovation (patent): Technological innovation is a key variable in resolving the contradiction between economic development and environmental pollution [40] and has a positive impact on GTFP, which is expressed in this paper as the number of domestic patent applications. Foreign direct investment (FDI)**:** FDI has a two-way impact on the green development of developing countries: On the one hand, FDI can use developing countries as “pollution haven”, thus worsening the environmental quality of the host country. On the other hand, FDI contributes to the improvement of GTFP in the host country through technology spillovers and relatively strict environmental standards, that is, the “pollution halo”. The variable definitions are shown in Table S4 (see in Supplementary Materials).

#### 2.2.3. Data Sources

The raw data in this paper come from the China Urban Statistical Yearbook, the China County Statistical Yearbook, the China Statistical Yearbook, the China Rural Statistical Yearbook and various local statistical yearbooks, and all of them can be found on the EPSDATA website (https://www.epsnet.com.cn/index.html#/Index, accessed on 1 May 2020). Considering the missing data of some cities, the following methods are used in this paper to ensure data coherence.

(1) Data at the county level were summed to the city level. (2) A linear fitting method was used to fill in the missing values. (3) The provincial data were decomposed to the city level based on the ratio of the city aggregate data to the province. (4) Samples that still had missing values were removed. In addition, as the DEA measurement of GTFP is more sensitive to abnormal data, this paper applies 1% tail reduction to the data before and after. Finally, in order to preserve as much sample size and data quality as possible, we obtain balanced panel data for a total of 6732 samples from 306 cities in China from 1996–2017. The descriptive statistics of this paper are shown in Table S5 (see in Supplementary Materials).

## 3. Spatial Panel Model Test

### 3.1. Unit Root and Cointegration Tests

In order to avoid the spurious regression of the model due to the non-stationarity of the data, this paper first conducted a panel unit root test on each variable. In this paper, the IPS test [41] and Fisher test [42] are used to conduct the unit root test. The results are shown in Table 1. All the variables are significant at a 1% significance level, indicating that all the variables are stationary and there is no pseudo-regression problem.

The Kao test [43], Pedroni test [44] and Westerlund test [45] are used to test for panel cointegration. The Kao test is a homogeneous panel cointegration test, and the Pedroni test and Westerlund test are heterogeneous panel cointegration tests. The results are shown in Table 2. All the statistics are significant except for the Westerlund test, where LnMis_L and LnMis_N are not significant at a 10% level of significance. To a large extent, this indicates that we can reject the original hypothesis of “H0: no cointegration relationship”.

### 3.2. Multicollinearity Test

In this paper, the model is also tested for multicollinearity using the variance inflation factor (VIF). The VIF is the ratio of the variance in the presence of multicollinearity between the explanatory variables to the variance in the absence of multicollinearity. When 0<VIF<10, there is no multicollinearity; when 10≤VIF<100, there is strong multicollinearity; and when VIF≥100, there is severe multicollinearity. As can be seen from the results in Table 3, the mean value of VIF is 2.19, which is much smaller than 10, so there is no need to worry about the existence of multicollinearity.

### 3.3. Spatial Autocorrelation Test

The existing studies generally use Moran’s I index to test for univariate spatial autocorrelation. According to You and Lv [46] and Anselin and Florax [47], a positive and significant Moran’s I index indicates spatial clustering among the sample areas, while a significantly negative Moran’s I index indicates spatial dispersion among the sample areas.

Table 4 shows the results of the spatial autocorrelation test for the GTFP of 306 cities in China from 1996–2017. As can be seen from the second column, the Moran’s I index was significantly positive at the 1% significance level from 1996–2017, with an overall value of 0.2767, indicating that there is a positive correlation between the GTFP of the region and the neighbor regions. However, the Moran’s I index showed a predominantly downward trend. It decreased from 0.3604 in 1996 to 0.1932 in 2017, indicating that the spatial clustering of GTFP between the local region and the neighbors has weakened year by year.

**Figure 2:**
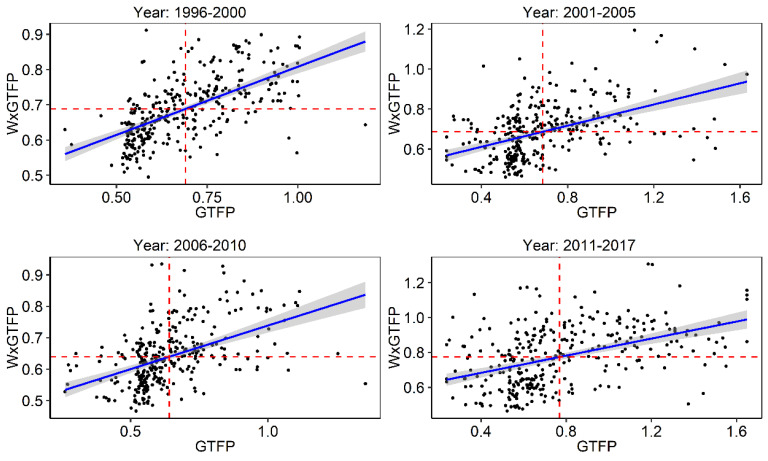
Global Moran scatter map of GTFP.

The Moran’s I test only describes the average level of spatial autocorrelation. If there is positive spatial autocorrelation in some regions and negative spatial autocorrelation in others, the two will cancel each other out, ultimately making the Moran’s I index an underestimate. Moran scatter map is used to further characterize the spatial autocorrelation between each region for the years 1996–2000, 2001–2005, 2006–2010 and 2011–2017. As can be seen from Figure 2, most of the regions are mainly clustered in the first and third quadrants, indicating that there is high–high and low–low clustering of GTFP in China, consistent with the aforementioned result that there is a significant positive spatial autocorrelation between regions.

### 3.4. Spatial Panel Model Diagnostic Test

To determine the fixed effect form of the model, the LR test is carried out, according to Li et al. [48]. As known from Table 5, both spatial and time fixed effects are significant at the 1% significance level, but the spatial fixed effect statistic is undoubtedly larger. Therefore, for the sake of making the empirical results more robust, the spatial fixed effect model is used in the subsequent empirical evidence.

In order to further determine which model is most suitable for SEM, SAR and SAREM, it is necessary to perform (robust) LM lag and (robust) LM error tests. Table 6 shows statistical tests based on the regression results of no fixed effect, spatial fixed effect, time fixed effect, and two-way fixed effect in space and time. It can be seen from the results that only the spatial fixed effect of LMLAG, R-LMLAG, LMERROR and R-LMERROR are all significant at the 1% significance level. It means that the SAREM model should be used. At the same time, it further shows that the use of the spatial fixed effect model is reasonable.

On the basis of clearly adopting the SAREM model, it is also necessary to determine whether the SDM should be adopted according to the LR and Wald tests. It can be seen from Table 7 that both LR and Wald tests reject the hypothesis of $H_{0}:\theta =0$, indicating that the SDM should be used in this paper. In addition, the statistics of the Hausman test are all significant at the 1% level, indicating that a fixed effect model should be used. Based on the above model identification test, it is finally determined that this paper should adopt the spatial Durbin model (SDM) that includes spatial lag and spatial error and, at the same time, control the city fixed effect.

## 4. Results and Discussion

### 4.1. GTFP Depicts

**Figure 3:**
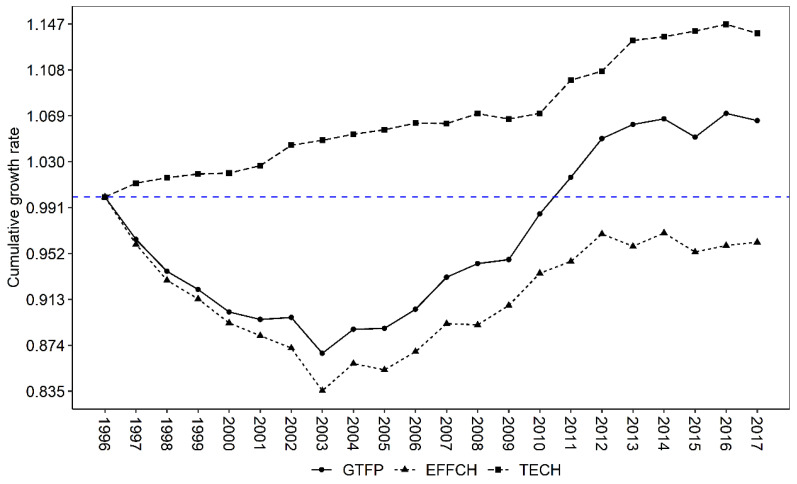
Cumulative change in average GTFP, EFFCH and TECH, 1996–2017. The blue dash line denotes 0 value horizontal line.

The average annual growth rate of China’s agricultural GTFP from 1996 to 2017 was 0.58%, which is very close to the 0.49–0.63 measured by Chen et al. [22] and 0.55 measured by Li et al. [49], indicating that the measurement results in this paper are robust. In addition, this paper decomposes GTFP change into technical efficiency change (EFFCH) and technological change (TECH). As can be seen from Figure 3, during the period of 1996–2017, EFFCH has shown a continuous upward trend and TECH has gone through three stages of decline (1996–2003), rise (2003–2014) and plateau (2014–2017).

The period 1996–2003 was a stage of decay, with an average growth rate of −1.9%, mainly due to the significant deterioration in EFFCH and the lack of TECH. The reasons mainly come from three aspects: Firstly, this paper uses sequential DEA, which does not allow for technological decline and treats technological progress as a smooth process, thus attributing all the reasons for the decline and sharp fluctuations in GTFP to technical inefficiency and variability [31]. Secondly, numerous studies have shown that the lack of agricultural technology diffusion in China, as well as the small-scale and fine-grained farmland management pattern, are the primary factors contributing to the lack of GTFP growth [36,50]. Thirdly, the reduction in the total sown area from 1998 to 2003 was due to ecological restoration and farmland restructuring. The period 2003–2014 was a rising stage of agricultural GTFP, with an average growth rate of 2.17%, mainly due to the upward acceleration of TECH while EFFCH decay slowed. This was followed by an adjustment phase from 2014–2017, with an average growth rate of 0.55%. This is mainly due to the fact that TECH showed an upward levelling off during the period of 2014–2017, while EFFCH did not improve sufficiently.

### 4.2. Benchmark Results

Table 8 shows the results of the benchmark regression. The variables (1)–(4) are the regression results of the SAREM model and (5)–(8) are the regression results of the SDM. As can be seen from the results, the coefficients of LnMis in (1)–(8) are negative and significant at the 1% statistical level, indicating that the deterioration of resource misallocation will significantly inhibit the increase in GTFP in agriculture. In terms of the size of the coefficients, the regression coefficients of the SAREM model will underestimate the negative effect of LnMis on GTFP. Based on this, only the regression results of SDM are interpreted in the subsequent empirical evidence in this paper, namely, (5)–(8).

In (5)–(8), the coefficients of LnMis (LnMis_L, LnMis_N, LnMis_M and LnMis_F) are significantly negative, but the coefficients of W×Ln_Mis_N and W×Ln_Mis_F are significantly positive. This suggests that whatever resource misallocation occurs, it will hinder the achievement of green growth in agriculture in the region and also that the occurrence of labor and fertilizer misallocation in the periphery has a significant contributing effect on GTFP. However, it should be noted that because the regression coefficient contains a feedback effect, that is, the influence of the local region on the surrounding regions, then the surrounding regions have an adverse effect on the local region. Therefore, understanding the marginal effect of resource misallocation on the impact of the local area requires subsequent effect decomposition.

In addition, we find that the control variables also have an impact on agricultural GTFP. The coefficients of LnRPP and W×LnRPP are positive, but neither is significant at the 10% statistical level. According to Hu et al. [37], there is a substitution relationship between labor and fertilizer, with farmers preferring to use fertilizer to secure food production when there is a shortage of labor, and, conversely, increasing labor capital ratios will promote progress in fertilizer-saving technologies, thereby improving agroecological conditions. However, excess agricultural labor will lead to land fragmentation and labor inefficiency to the detriment of total factor productivity in agriculture. These two opposing forces cancel each other out, making the effect of LnRPP on GTFP insignificant. The coefficient on LnPGDP is positive and significant at the 1% statistical level, indicating that local urban economic development can contribute to an increase in local agricultural GTFP. However, W×LnPGDP is significantly negative, indicating that neighbor economic development has a dampening effect on local GTFP. The coefficients of LnPD are all significantly negative, indicating that any increase in local urban population density will significantly inhibit the increase in agricultural GTFP. The coefficient of LnER is not statistically significant, but the coefficient of W×LnER is significantly negative, indicating that an increase in the intensity of environmental regulations in the local region cannot significantly contribute to agricultural GTFP, while an increase in the neighbor intensity of environmental regulations can negatively affect local agricultural GTFP in the local region. The coefficient of LnPatent is positive, but the coefficient of W×LnPatent is significant, indicating that local technological innovation can promote local agricultural GTFP. In contrast, technological innovation in other regions significantly suppresses local agricultural GTFP. The coefficients for both LnFDI and W×LnFDI are significant but in opposite directions. The former is positive and the latter is negative. This indicates that the increase in local investment in the region can effectively promote the increase in local agricultural GTFP. In contrast, the increase in FDI in the neighbor regions will significantly reduce the local agricultural GTFP.

### 4.3. Robustness Test

To test the robustness of the estimation results, this paper uses a spatial weight matrix based on the square of the inverse of the geographical distance:

(19) WD=Wij={1dij2, i≠j0, i=j

where $W_{\mathrm{ij}}$ is the weight of geographical distance between the center of region i and j and $d_{\mathrm{ij}}$ is the linear distance, 1dij2 is the reciprocal of the square of the linear distance between two regions. When the distance between region i and region j is relatively close, the larger is $W_{\mathrm{ij}}$, and the other is the smaller.

It can be seen from Table 9 that the coefficients of LnMis are significantly negative, indicating that changing the spatial weight matrix did not affect the results of the key variables in this paper. In other words, the benchmark results in this paper are robust. In addition, in (1)–(4), the coefficient of W×LnMis is significantly positive at the 5% statistical level, and the size of the coefficient has also increased. This is because the spatial geographic distance weight matrix not only considers the influence of areas with common borders, but also takes into account the influence of non-adjacent areas. For example, areas in the same province but not adjacent to each other still have spatial spillover effects. This also shows that compared with the spatial adjacency matrix, the spatial distance weight matrix can more truly reflect the spillover effect between regions. Therefore, in the follow-up empirical research, this paper mainly reports the results of the spatial distance weight matrix.

### 4.4. Effect Decomposition Results

The coefficients of the SDM do not directly reflect the marginal effects of the explanatory variables on the dependent variable [48] and need to be further decomposed into direct, indirect and total effects. The direct effect represents the net effect of changes in the local dependent on the local GTFP, the indirect effect represents the effect of changes in the local dependent variable in other regions on the local GTFP and the total effect is the sum of the direct and indirect effects.

As can be seen from Table 10, the coefficients of direct effects of LnMis are significantly negative in (1)–(4), indicating that whatever resource misallocation occurs, it will significantly inhibit the green growth of agriculture in the region. In terms of impact size, land misallocation has the greatest negative impact, followed by fertilizer misallocation, labor misallocation and machinery misallocation. The reason for this is that in a situation where land is difficult to operate on a large scale, farmers are more inclined to secure yields by applying excessive fertilizers, which not only makes fertilizer utilization inefficient [36] but also degrades the agroecological environment [35,36,51,52]. The coefficients of indirect effects of LnMis are all positive, but only the coefficient of (2) is statistically significant at 10%, indicating that the neighbor labor misallocation can significantly contribute to the local GTFP. In recent years, with the large-scale transfer of rural labor to economically developed regions, it is conducive to alleviating the contradiction between rural people and land. However, the transferred labor is mainly young and middle-aged labor, which will cause a labor shortage in the outflowing regions, which is not conducive to the local agriculture development. On the contrary, for those places where labor is transferred, the labor can contribute to the regional economic development. With the direct and indirect effects offsetting each other, the coefficients of the total effects are insignificant.

### 4.5. Heterogeneity Test

The differences in the economic development, the marketisation and the climate are responsible for Chinese agricultural development’s obvious regional characteristics. In this paper, the sample is divided into three sub-sample groups according to the eastern, central and western parts of the country, in accordance with the usual practice of the academic community, and the regressions are conducted separately.

As can be seen from Table 11, the coefficients of resource misallocation (LnMis_L, LnMis_N, LnMis_M, LnMis_F) are significantly negative in the eastern, central and western sample groups, indicating that the inhibiting effect of resource misallocation on GTFP does not vary according to regional differences.

In Table 12, the direct effect of LnMis_L is significantly negative, indicating that land misallocation can significantly reduce GTFP in the region. The direct and indirect effects of LnMis_N, LnMis_M and LnMis_F are all significantly negative. It is noteworthy that the indirect effects are smaller than the direct effects in the central and western regions, except for the eastern region where the indirect effects are larger than the direct effects. This indicates that GTFP in the eastern region is more susceptible to negative spillovers from resource misallocation in other regions, while the central and western regions are mainly affected by local resource misallocation. The reasons for this may come from the following: Firstly, the degree of factor marketisation in the eastern region is relatively higher, and the degree of resource misallocation is also lower; thus, the negative impact of the local resource misallocation on the local total factor productivity is also relatively smaller. Secondly, compared to the central and western regions, environmental regulations are stricter in the eastern region, which can mitigate the negative impact of resource misallocation on environmental pollution. Thirdly, agricultural non-point source pollution is diffuse, and there is a strong spillover effect of resource misallocation on the agricultural ecological environment in the other regions.

### 4.6. Geographical Distance Dynamic Test

Additionally, this paper uses 300 km as the benchmark to construct a spatial distance weight matrix, and then measures the spatial spillover effect under different geographical distance thresholds according to the increasing distance of 300 km.

As can be seen from Table 13, the coefficients of LnMis (LnMis_L, LnMis_N, LnMis_M and LnMis_F) are all significantly negative, indicating that the negative effect of LnMis on GTFP does not change depending on the spatial geographical distance. In terms of coefficient magnitude, the coefficients of LnMis_L, LnMis_M and LnMis_F gradually increase between 300–1500 km and then gradually decrease. The coefficients of WLnMis (W×LnMis_L, W×LnMis_N, W×LnMis_M and W×LnMis_F) are all positive, and the significance and size of the coefficients tend to increase with increasing distance. This indicates that as the spatial distance increases, the more cities are included and the greater the positive spillover effect of resource misallocation.

**Figure 4:**
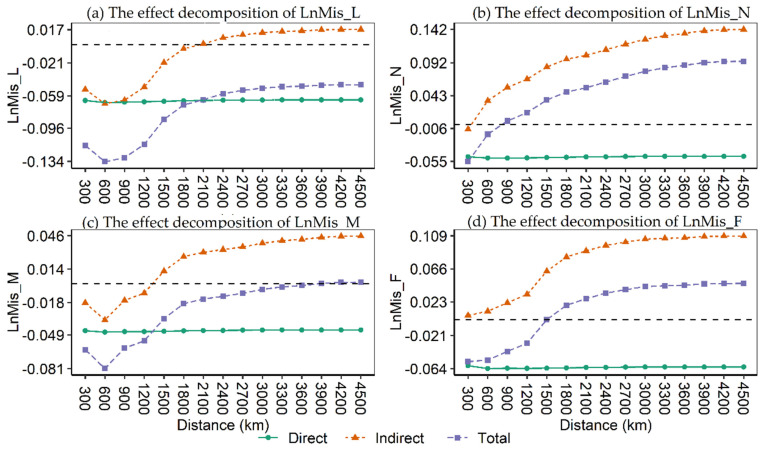
Direct, indirect and total effects with changing geographical distance. The black dash line denotes 0 value line.

Figure 4 shows the trends in the direct, indirect and total effects. Where Figure 4a shows the effect decomposition of LnMis_L, Figure 4b shows the effect decomposition of LnMis_N, Figure 4c shows the effect decomposition of LnMis_M and Figure 4d shows the effect decomposition of LnMis_F. As can be seen from the figure, the direct effect is almost parallel to the horizontal axis, which is largely due to the fact that the calculation of the direct effect is mainly determined by the coefficients of LnMis and has little to do with the spatial distance weight matrix. In contrast, the indirect effect rises with increasing spatial distance, thus driving the total effect to also show an upward trend. This corroborates the above inference that the positive spillover effect of resource misallocation increases with increasing spatial distance and the number of cities included.

## 5. Conclusions

This paper empirically examines the spatial spillover effects of resource misallocation affecting agricultural GTFP, based on balanced panel data from 306 cities in China from 1996–2017 using a spatial panel model. The main findings of this paper are summarized as follows.

Firstly, Chinese agricultural GTFP is generally characterized by a “U” shaped change, with a decline (between 1996–2003) followed by an increase (between 2003–2017). Secondly, local resource misallocation hinders the achievement of green growth in agriculture, but labor misallocation in neighbor regions has a significant contribution to GTFP. The heterogeneity test results show that GTFP in the eastern region is more susceptible to negative spillovers from resource misallocation than in other regions, while the decline in GTFP in the central and western regions is noted to be affected by resource misallocation in the region. Finally, the negative effect of local resource misallocation on GTFP does not change depending on spatial geographical distance. In contrast, the indirect effect rises with increasing spatial distance, thus driving the total effect to also show an upward trend.

Based on the above conclusions, the policy implications of this paper are as follows. (1) Chinese government should strengthen agricultural green technology innovation and diffusion. The empirical results show that Chinese agricultural technological progress has shown a continuous upward trend, but the technical efficiency has always been in a deteriorating trend, thus leading to the weak growth of Chinese agricultural GTFP. Therefore, while the Chinese government should increase the research and development of green technologies in agriculture, it should also strengthen the promotion of green technologies and enhance the training of farmers to apply the new technologies in agricultural production. (2) Chinese government should strengthen environmental regulation. In this paper, the excessive use of chemical fertilizer and improper disposal of agricultural straw are the main causes of environmental pollution. Tang et al. [53] argued that non-point source pollution caused about 6% of agricultural GDP loss. Based on this, on the one hand, the Chinese government should strengthen the propaganda and technical guidance on scientific fertilizer application and promote soil testing and formula technology. On the other hand, local governments in China should stop the burning and abandonment of agricultural straw and introduce new technologies for the comprehensive use of agricultural straw, such as feed and organic fertilizer. (3) Chinese government should promote the free movement of labor between regions and sectors. In this paper, resource (labor, land, machinery and fertilizer) misallocation will inhibit the agricultural GTFP. Therefore, it is urgent to correct the misallocation and promote the effective allocation of resource and factors. Among them, labor is the most important input, because if the rural and agricultural sectors gather a large amount of labor, it will not only lead to labor misallocation but also make it impossible to transfer and concentrate the cultivated land, achieve a large-scale operation, and then cause the misallocation of land, machinery and fertilizer. On the one hand, the Chinese government should eliminate the urban–rural dual structure, realize the equalization of urban and rural public services, and promote the flow of rural surplus labor to cities; on the other hand, the Chinese government should strengthen skills training in rural areas, so that farmers have the skills required to engage in non-agricultural work.

This paper has some limitations, which are as follows: Firstly, in the research sample, this study uses city data, which can directly reflect the temporal and spatial differences of agricultural resource misallocation and GTFP. However, the city data will smooth out the differences in farmers’ preference factors, and it is difficult to reveal the micro-mechanisms that the effect of resource misallocation will have on GTFP. In the future, sample data at the level of farmers or agricultural products can be used to explore in depth the micro-mechanism. Secondly, the Chinese government promises to strive to achieve peak CO_2_ emissions by 2030 and carbon neutrality by 2060. Although industry is the source of greenhouse gases, rapid development of agriculture also plays an important role, and the carbon emissions caused each year should not be underestimated. In the future, we will consider the relationship between resource misallocation and agricultural carbon emissions.

## Figures and Tables

**Table 1:** Panel unit root tests results.

Variable	IPS Test	P Test	Inverse Normal Test	Logit Test	Modified P Test
GTFP	−7.6441 ***	2031.9801 ***	−29.2566 ***	−30.9081 ***	40.5874 ***
LnMis_L	−12.3208 ***	2246.0403 ***	−31.7215 ***	−34.4199 ***	46.7059 ***
LnMis_N	−12.1691 ***	2229.5186 ***	−31.5401 ***	−34.1267 ***	46.2337 ***
LnMis_M	−12.3316 ***	2327.0760 ***	−33.0143 ***	−35.8447 ***	49.0222 ***
LnMis_F	−13.3892 ***	2322.1941 ***	−32.8411 ***	−35.8331 ***	48.8826 ***
LnRPP	−16.6487 ***	3059.5790 ***	−39.9604 ***	−47.5250 ***	69.9594 ***
LnPGDP	−16.9106 ***	3048.1963 ***	−37.2356 ***	−46.9011 ***	69.6340 ***
LnPD	−24.1352 ***	2789.6737 ***	−37.9656 ***	−43.5162 ***	62.2447 ***
LnER	−19.4832 ***	1854.5856 ***	−26.1719 ***	−27.6148 ***	35.5169 ***
LnPatent	2.8011	1366.4640 ***	−20.3258 ***	−19.6744 ***	21.5649 ***
LnFDI	−12.0636 ***	1935.5231 ***	−27.5660 ***	−29.0614 ***	37.8304 ***

*** *p* < 0.01.

**Table 2:** Panel cointegration tests results.

Method	LnMis_L		LnMis_N		LnMis_M		LnMis_F	
	Statistic	p	Statistic	p	Statistic	p	Statistic	p
**Kao test**								
Modified Dickey–Fuller t	−11.092	0.000	−10.739	0.000	−10.691	0.000	−11.222	0.000
Dickey–Fuller t	−12.948	0.000	−12.700	0.000	−12.655	0.000	−13.055	0.000
Augmented Dickey–Fuller t	−18.191	0.000	51.831	0.000	62.361	0.000	56.242	0.000
Unadjusted modified Dickey–Fuller t	−18.320	0.000	−17.814	0.000	−17.801	0.000	−18.400	0.000
Unadjusted Dickey–Fuller t	−16.041	0.000	−15.764	0.000	−15.739	0.000	−16.113	0.000
**Pedroni test**								
Modified Phillips–Perron t	21.781	0.000	21.823	0.000	21.867	0.000	21.727	0.000
Phillips–Perron t	−14.018	0.000	−14.583	0.000	−13.882	0.000	−14.845	0.000
Augmented Dickey–Fuller t	−11.025	0.000	−11.232	0.000	−10.469	0.000	−11.167	0.000
**Westerlund test**								
Variance ratio	1.243	0.107	1.069	0.143	1.843	0.033	1.615	0.053

**Table 3:** Multicollinearity test results.

Variable	LnMis_L		LnMis_N		LnMis_M		LnMis_F	
	VIF	1/VIF	VIF	1/VIF	VIF	1/VIF	VIF	1/VIF
LnMis	1.105	0.905	1.079	0.927	1.117	0.895	1.100	0.909
LnRPP	1.692	0.591	1.692	0.591	1.693	0.591	1.693	0.591
LnPGDP	1.368	0.731	1.366	0.732	1.365	0.732	1.368	0.731
LnPD	1.336	0.749	1.334	0.750	1.342	0.745	1.337	0.748
LnER	2.728	0.367	2.717	0.368	2.710	0.369	2.715	0.368
LnPatent	4.312	0.232	4.317	0.232	4.317	0.232	4.315	0.232
LnFDI	2.812	0.356	2.814	0.355	2.852	0.351	2.821	0.354
Mean VIF	2.193		2.189		2.199		2.193	

**Table 4:** Global Moran’s I statistical indexes of 306 cities.

Year	Moran’s I	Expectation	Std. Dev.	z-Value	*p*-Value
1996	0.3604 ***	−0.00323	0.0260	13.8346	0.0000
1997	0.3826 ***	−0.00323	0.0260	14.6998	0.0000
1998	0.3846 ***	−0.00323	0.0260	14.8185	0.0000
1999	0.3344 ***	−0.00323	0.0260	12.8724	0.0000
2000	0.3102 ***	−0.00323	0.0259	11.9542	0.0000
2001	0.2985 ***	−0.00323	0.0259	11.5063	0.0000
2002	0.3107 ***	−0.00323	0.0260	11.9687	0.0000
2003	0.2333 ***	−0.00323	0.0259	9.0131	0.0000
2004	0.2140 ***	−0.00323	0.0259	8.2633	0.0000
2005	0.2309 ***	−0.00323	0.0259	8.9097	0.0000
2006	0.2077 ***	−0.00323	0.0259	8.0128	0.0000
2007	0.2348 ***	−0.00323	0.0259	9.0466	0.0000
2008	0.2271 ***	−0.00323	0.0260	8.7500	0.0000
2009	0.2354 ***	−0.00323	0.0260	9.0678	0.0000
2010	0.2288 ***	−0.00323	0.0260	8.8068	0.0000
2011	0.2400 ***	−0.00323	0.0260	9.2372	0.0000
2012	0.2421 ***	−0.00323	0.0260	9.3179	0.0000
2013	0.2167 ***	−0.00323	0.0260	8.3387	0.0000
2014	0.2118 ***	−0.00323	0.0260	8.1480	0.0000
2015	0.1831 ***	−0.00323	0.0260	7.0435	0.0000
2016	0.1876 ***	−0.00323	0.0260	7.2160	0.0000
2017	0.1932 ***	−0.00323	0.0260	7.4336	0.0000
1996–2017	0.2767 ***	−0.00323	0.0056	49.1922	0.0000

*** *p* < 0.01.

**Table 5:** Spatial and time fixed effects test results.

Test Statistics	LnMis_L	LnMis_N	LnMis_M	LnMis_F
Spatial fixed effect LR-test	9668.304 ***	9642.018 ***	9593.892 ***	9671.523 ***
Time fixed effect LR-test	620.769 ***	624.262 ***	617.416 ***	622.755 ***

*** *p* < 0.01.

**Table 6:** Spatial diagnostic test results.

Effect	Variable	LMLAG	R-LMLAG	LMERROR	R-LMERROR
**No fixed effect**	LnMis_L	9.259 ***	40.444 ***	0.001	31.186 ***
	LnMis_N	7.371 ***	38.699 ***	0.071	31.399 ***
	LnMis_M	6.664 ***	36.615 ***	0.149	30.100 ***
	LnMis_F	7.591 ***	36.874 ***	0.025	29.309 ***
**Space fixed effect**	LnMis_L	238.822 ***	184.617 ***	129.156 ***	74.952 ***
	LnMis_N	236.288 ***	200.390 ***	124.005 ***	88.106 ***
	LnMis_M	239.304 ***	185.106 ***	130.653 ***	76.455 ***
	LnMis_F	246.030 ***	184.546 ***	135.612 ***	74.128 ***
**Time fixed effect**	LnMis_L	52.819 ***	5.950 **	47.947 ***	1.079
	LnMis_N	47.815 ***	4.350 **	45.044 ***	1.580
	LnMis_M	45.461 ***	5.287 **	41.184 ***	1.010
	LnMis_F	48.461 ***	4.131 **	46.199 ***	1.869
**Time-space fixed effect**	LnMis_L	1.858	17.070 ***	0.228	15.440 ***
	LnMis_N	1.424	30.476 ***	0.004	29.056 ***
	LnMis_M	1.843	17.645 ***	0.246	16.048 ***
	LnMis_F	2.137	13.866 ***	0.436	12.166 ***

*** *p* < 0.01, ** *p* < 0.05.

**Table 7:** LR, Wald and Hausman test results.

Test Statistics	LnMis_L	LnMis_N	LnMis_M	LnMis_F
Wald spatial lag	38.342 ***	39.811 ***	32.250 ***	45.317 ***
LR spatial lag	38.202 ***	39.657 ***	32.157 ***	45.115 ***
Wald spatial error	34.032 ***	35.705 ***	27.244 ***	41.846 ***
LR spatial error	33.804 ***	35.481 ***	27.097 ***	27.097 ***
Wald spatial lag and spatial error	43.956 ***	45.644 ***	38.603 ***	48.646 ***
LR spatial lag and spatial error	43.815 ***	45.470 ***	38.494 ***	38.494 ***
Hausman test	140.460 ***	186.880 ***	94.516 ***	108.440 ***

*** *p* < 0.01.

**Table 8:** Benchmark model results.

Variable	(1)	(2)	(3)	(4)	(5)	(6)	(7)	(8)
	LnMis_L	LnMis_N	LnMis_M	LnMis_F	LnMis_L	LnMis_N	LnMis_M	LnMis_F
LnMis	−0.054 ***	−0.046 ***	−0.041 ***	−0.053 ***	−0.060 ***	−0.049 ***	−0.041 ***	−0.058 ***
	(0.005)	(0.006)	(0.005)	(0.005)	(0.006)	(0.006)	(0.005)	(0.005)
LnRPP	0.001	0.005	0.005	0.001	0.009	0.009	0.009	0.009
	(0.005)	(0.005)	(0.005)	(0.005)	(0.006)	(0.006)	(0.006)	(0.006)
LnPGDP	0.045 ***	0.041 ***	0.041 ***	0.044 ***	0.051 ***	0.051 ***	0.052 ***	0.050 ***
	(0.004)	(0.004)	(0.004)	(0.004)	(0.005)	(0.005)	(0.005)	(0.005)
LnPD	−0.002 ***	−0.005 ***	−0.004 ***	−0.002 ***	−0.004 ***	−0.004 ***	−0.003 ***	−0.003 ***
	(0.001)	(0.001)	(0.001)	(0.001)	(0.001)	(0.001)	(0.001)	(0.001)
LnER	−0.025 ***	−0.050 ***	−0.051 ***	−0.025 ***	−0.0003	−0.001	−0.009	−0.005
	(0.007)	(0.006)	(0.006)	(0.007)	(0.015)	(0.015)	(0.015)	(0.015)
LnPatent	0.019 ***	−0.008 ***	−0.008 ***	0.020 ***	0.014	0.017*	0.017 **	0.016 *
	(0.003)	(0.002)	(0.002)	(0.003)	(0.009)	(0.009)	(0.009)	(0.009)
LnFDI	0.018 ***	0.011 ***	0.010 ***	0.019 ***	0.029 ***	0.027 ***	0.027 ***	0.031 ***
	(0.004)	(0.004)	(0.004)	(0.004)	(0.008)	(0.008)	(0.008)	(0.008)
W × Ln_Mis					0.003	0.028 **	−0.007	0.028 **
					(0.014)	(0.014)	(0.013)	(0.013)
W × LnRPP					−0.014	−0.014	−0.015	−0.014
					(0.009)	(0.009)	(0.009)	(0.009)
W × LnPGDP					−0.019 **	−0.017 **	−0.018 **	−0.015 **
					(0.007)	(0.007)	(0.007)	(0.007)
W × LnPD					−0.002	−0.001	−0.002	−0.002
					(0.001)	(0.001)	(0.001)	(0.001)
W × LnER					−0.060 ***	−0.060 ***	−0.050 ***	−0.056 ***
					(0.018)	(0.018)	(0.018)	(0.018)
W × LnPatent					−0.016 *	−0.020 **	−0.021 **	−0.019 **
					(0.009)	(0.009)	(0.009)	(0.009)
W × LnFDI					−0.025 ***	−0.022 **	−0.021 **	−0.026 ***
					(0.009)	(0.009)	(0.009)	(0.009)
λ	−0.847 ***	0.698 ***	0.695 ***	−0.850 ***	0.691 ***	0.700 ***	0.696 ***	0.693 ***
	(0.041)	(0.018)	(0.018)	(0.041)	(0.019)	(0.018)	(0.019)	(0.019)
ρ	0.782 ***	−0.788 ***	−0.775 ***	0.784 ***	−0.766 ***	−0.792 ***	−0.779 ***	−0.765 ***
	(0.015)	(0.045)	(0.045)	(0.015)	(0.046)	(0.045)	(0.046)	(0.046)
City	Yes	Yes	Yes	Yes	Yes	Yes	Yes	Yes
N	6426	6426	6426	6426	6426	6426	6426	6426

The parenthesis represents the standard error values, *** *p* < 0.01, ** *p* < 0.05, * *p* < 0.1.

**Table 9:** Robustness test results.

Variable	(1)	(2)	(3)	(4)
	LnMis_L	LnMis_N	LnMis_M	LnMis_F
LnMis	−0.064 ***	−0.050 ***	−0.045 ***	−0.064 ***
	(0.006)	(0.006)	(0.005)	(0.006)
W×LnMis	0.052 **	0.074 ***	0.045 **	0.076 ***
	(0.022)	(0.020)	(0.019)	(0.021)
λ	0.741 ***	0.747 ***	0.745 ***	0.739 ***
	(0.024)	(0.024)	(0.024)	(0.024)
ρ	−0.670 ***	−0.685 ***	−0.680 ***	−0.665 ***
	(0.053)	(0.053)	(0.053)	(0.053)
Control	Yes	Yes	Yes	Yes
W × Control	Yes	Yes	Yes	Yes
City	Yes	Yes	Yes	Yes
N	6426	6426	6426	6426

The parenthesis represents the standard error values, *** *p* < 0.01, ** *p* < 0.05.

**Table 10:** Effect decomposition results.

Variable	(1)	(2)	(3)	(4)
	LnMis_L	LnMis_N	LnMis_M	LnMis_F
**Direct effect**	−0.063 ***	−0.048 ***	−0.044 ***	−0.062 ***
	(0.006)	(0.006)	(0.005)	(0.006)
**Indirect effect**	0.017	0.142 *	0.046	0.109
	(0.083)	(0.084)	(0.071)	(0.071)
**Total effect**	−0.046	0.094	0.002	0.047
	(0.085)	(0.086)	(0.071)	(0.074)

The parenthesis represents the standard error values, *** *p* < 0.01, * *p* < 0.1.

**Table 11:** Heterogeneity test results.

Variable	(1)	(2)	(3)	(4)
	LnMis_L	LnMis_N	LnMis_M	LnMis_F
**Eastern**				
LnMis	−0.080 ***	−0.033 ***	−0.040 ***	−0.067 ***
	(0.011)	(0.010)	(0.009)	(0.010)
W × LnMis	0.001	0.026	0.005	0.009
	(0.021)	(0.019)	(0.020)	(0.020)
λ	−0.552 ***	−0.569 ***	−0.556 ***	−0.559 ***
	(0.059)	(0.059)	(0.060)	(0.059)
ρ	0.674 ***	0.673 ***	0.669 ***	0.676 ***
	(0.029)	(0.029)	(0.029)	(0.029)
City	Yes	Yes	Yes	Yes
N	2289	2289	2289	2289
**Central**				
LnMis	−0.035 ***	−0.049 ***	−0.046 ***	−0.048 ***
	(0.010)	(0.010)	(0.010)	(0.010)
W × LnMis	0.023	−0.014	0.026	0.010
	(0.018)	(0.020)	(0.018)	(0.018)
λ	0.504 ***	0.493 ***	0.504 ***	0.496 ***
	(0.043)	(0.044)	(0.043)	(0.044)
ρ	−0.366 ***	−0.351 ***	−0.370 ***	−0.356 ***
	(0.069)	(0.070)	(0.068)	(0.069)
City	Yes	Yes	Yes	Yes
N	2121	2121	2121	2121
**Western**				
LnMis	−0.085 ***	−0.063 ***	−0.041 ***	−0.078 ***
	(0.010)	(0.010)	(0.008)	(0.009)
W × LnMis	0.008	0.064 ***	0.017	0.050 ***
	(0.019)	(0.019)	(0.015)	(0.017)
λ	0.274 ***	0.294 ***	0.284 ***	0.262 ***
	(0.065)	(0.065)	(0.070)	(0.069)
ρ	−0.101	−0.126	−0.105	−0.080
	(0.083)	(0.085)	(0.089)	(0.087)
City	Yes	Yes	Yes	Yes
N	2016	2016	2016	2016

The parenthesis represents the standard error values, *** *p* < 0.01.

**Table 12:** Heterogeneity tests: Effect decomposition.

Variable	Eastern			Central			Western		
	(1)	(2)	(3)	(4)	(5)	(6)	(7)	(8)	(9)
	Direct	Indirect	Total	Direct	Indirect	Total	Direct	Indirect	Total
LnMis_L	−0.084 ***	0.033 **	−0.051 ***	−0.035 ***	0.009	−0.025	−0.086 ***	−0.021	−0.107 ***
	(0.011)	(0.016)	(0.014)	(0.01)	(0.032)	(0.036)	(0.01)	(0.022)	(0.026)
LnMis_N	−0.037 ***	0.032 **	−0.005	−0.054 ***	−0.071 *	−0.125 ***	−0.060 ***	0.061**	0.000
	(0.01)	(0.015)	(0.012)	(0.011)	(0.037)	(0.042)	(0.011)	(0.024)	(0.027)
LnMis_M	−0.043 ***	0.020	−0.022	−0.045 ***	0.005	−0.040	−0.040 ***	0.007	−0.033
	(0.009)	(0.014)	(0.014)	(0.011)	(0.039)	(0.044)	(0.008)	(0.022)	(0.025)
LnMis_F	−0.072 ***	0.034 **	−0.038 **	−0.050 ***	−0.027	−0.077 *	−0.076 ***	0.038*	−0.038
	(0.011)	(0.015)	(0.015)	(0.011)	(0.037)	(0.043)	(0.011)	(0.021)	(0.024)

The parenthesis represents the standard error values, *** *p* < 0.01, ** *p* < 0.05, * *p* < 0.1.

**Table 13:** Geographical distance dynamic test results.

Dis. (km)	LnMis_L	W × LnMis_L	LnMis_N	W × LnMis_N	LnMis_M	W × LnMis_M	LnMis_F	W × LnMis_F
300	−0.0607 ***	0.0111	−0.0477 ***	0.0244 **	−0.0436 ***	0.0171	−0.0604 ***	0.0375 ***
600	−0.0638 ***	0.0156	−0.0514 ***	0.0462 ***	−0.0449 ***	0.0164	−0.0641 ***	0.0454 ***
900	−0.0642 ***	0.0234	−0.0517 ***	0.0533 ***	−0.0452 ***	0.0262 *	−0.0641 ***	0.0511 ***
1200	−0.0644 ***	0.0305	−0.0515 ***	0.0567 ***	−0.0455 ***	0.0296 *	−0.0644 ***	0.0553 ***
1500	−0.0646 ***	0.0399 **	−0.0512 ***	0.0615 ***	−0.0457 ***	0.0363 **	−0.0647 ***	0.0646 ***
1800	−0.0644 ***	0.0449 **	−0.0509 ***	0.0643 ***	−0.0456 ***	0.0403 **	−0.0645 ***	0.0698 ***
2100	−0.0641 ***	0.0466 **	−0.0505 ***	0.0653 ***	−0.0453 ***	0.0414 **	−0.0642 ***	0.0717 ***
2400	−0.0641 ***	0.0487 **	−0.0503 ***	0.0672 ***	−0.0452 ***	0.0421 **	−0.0641 ***	0.0734 ***
2700	−0.0639 ***	0.0498 **	−0.0501 ***	0.0689 ***	−0.0451 ***	0.0427 **	−0.0638 ***	0.0743 ***
3000	−0.0638 ***	0.0506 **	−0.0500 ***	0.0706 ***	−0.0450 ***	0.0436 **	−0.0637 ***	0.0751 ***
3300	−0.0637 ***	0.0510 **	−0.0500 ***	0.0718 ***	−0.0449 ***	0.0442 **	−0.0637 ***	0.0753 ***
3600	−0.0637 ***	0.0513 **	−0.0500 ***	0.0725 ***	−0.0449 ***	0.0445 **	−0.0636 ***	0.0754 ***
3900	−0.0637 ***	0.0517 **	−0.0501 ***	0.0734 ***	−0.0449 ***	0.0450 **	−0.0637 ***	0.0758 ***
4200	−0.0637 ***	0.0518 **	−0.0501 ***	0.0739 ***	−0.0450 ***	0.0453 **	−0.0637 ***	0.0759 ***
4500	−0.0637 ***	0.0518 **	−0.0501 ***	0.0739 ***	−0.0450 ***	0.0453 **	−0.0637 ***	0.0759 ***

*** *p* < 0.01, ** *p* < 0.05, * *p* < 0.1.

## Data Availability

The data that support the finding of this study are available from the corresponding author upon reasonable request.

## Supplementary Materials

The following supporting information can be downloaded at: https://www.mdpi.com/article/10.3390/ijerph192315718/s1, Table S1: Fertilizer loss rate in different regions; Table S2: Pollution production coefficient of main crops (10^−5^t/t); Table S3: Nutrient loss rate of agricultural waste in different provinces (%); Table S4: Variables and definition; Table S5: Descriptive statistics of variables.

## References

[1] Fei R., Lin B. (2016). Energy efficiency and production technology heterogeneity in China’s agricultural sector: A meta-frontier approach. Technol. Forecast. Soc..

[2] Xu B., Chen W., Zhang G., Wang J., Ping W., Luo L., Chen J. (2020). How to achieve green growth in China’s agricultural sector. J. Clean. Prod..

[3] Sheng Y., Tian X., Qiao W., Peng C. (2020). Measuring agricultural total factor productivity in China: Pattern and drivers over the period of 1978–2016. Aust. J. Agric. Resour. Econ..

[4] Hallegatte S., Heal G., Fay M., Treguer D. (2011). From Growth to Green Growth—A Framework.

[5] Lin B., Chen Z. (2018). Does factor market distortion inhibit the green total factor productivity in China?. J. Clean. Prod..

[6] Ji Z. (2020). Does factor market distortion affect industrial pollution intensity? Evidence from China. J. Clean. Prod..

[7] Jones C. (2011). Misallocation, Economic Growth, and Input-Output Economics.

[8] Yang M., Yang F., Sun C. (2018). Factor market distortion correction, resource reallocation and potential productivity gains: An empirical study on China’s heavy industry sector. Energy Econ..

[9] Restuccia D., Rogerson R. (2017). The causes and costs of misallocation. J. Econ. Perspect..

[10] Ouyang X., Wei X., Sun C., Du G. (2018). Impact of factor price distortions on energy efficiency: Evidence from provincial-level panel data in China. Energy Policy.

[11] Su H., Liang B. (2021). The impact of regional market integration and economic opening up on environmental total factor energy productivity in Chinese provinces. Energy Policy.

[12] Tan R., Lin B., Liu X. (2019). Impacts of eliminating the factor distortions on energy efficiency—A focus on China’s secondary industry. Energy.

[13] Adamopoulos T., Brandt L., Leight J., Restuccia D. (2022). Misallocation, selection, and productivity: A quantitative analysis with panel data from China. Econometrica.

[14] Zhao X. (2020). Land and labor allocation under communal tenure: Theory and evidence from China. J. Dev. Econ..

[15] Yang Z., Shao S., Yang L., Miao Z. (2018). Improvement pathway of energy consumption structure in China’s industrial sector: From the perspective of directed technical change. Energy Econ..

[16] Yang Z., Shao S., Fan M., Yang L. (2021). Wage distortion and green technological progress: A directed technological progress perspective. Ecol. Econ..

[17] Tombe T., Winter J. (2015). Environmental policy and misallocation: The productivity effect of intensity standards. J. Environ. Econ. Manag..

[18] Bian Y., Song K., Bai J. (2019). Market segmentation, resource misallocation and environmental pollution. J. Clean. Prod..

[19] Zhang J., Wang J., Yang X., Ren S., Ran Q., Hao Y. (2021). Does local government competition aggravate haze pollution? A new perspective of factor market distortion. Socio-Econ. Plan. Sci..

[20] Wang S., Zhao D., Chen H. (2020). Government corruption, resource misallocation, and ecological efficiency. Energy Econ..

[21] Hao Y., Gai Z., Wu H. (2020). How do resource misallocation and government corruption affect green total factor energy efficiency? Evidence from China. Energy Policy.

[22] Chen Y., Miao J., Zhu Z. (2021). Measuring green total factor productivity of China’s agricultural sector: A three-stage SBM-DEA model with non-point source pollution and CO_2_ emissions. J. Clean. Prod..

[23] Xu M., Tan R. (2021). Removing energy allocation distortion to increase economic output and energy efficiency in China. Energy Policy.

[24] Han H., Zhong Z., Wen C., Sun H. (2018). Agricultural environmental total factor productivity in China under technological heterogeneity: Characteristics and determinants. Environ. Sci. Pollut. Res..

[25] Han H., Ding T., Nie L., Hao Z. (2020). Agricultural eco-efficiency loss under technology heterogeneity given regional differences in China. J. Clean. Prod..

[26] Liu Y., Feng C. (2019). What drives the fluctuations of “green” productivity in China’s agricultural sector? A weighted Russell directional distance approach. Resour. Conserv. Recycl..

[27] Tang K., Hailu A., Yang Y. (2020). Agricultural chemical oxygen demand mitigation under various policies in China: A scenario analysis. J. Clean. Prod..

[28] Zou L., Liu Y., Wang Y., Hu X. (2020). Assessment and analysis of agricultural non-point source pollution loads in China: 1978–2017. J. Environ. Manag..

[29] Qu Y., Zhang Q., Zhan L., Jiang G., Si H. (2022). Understanding the nonpoint source pollution loads’ spatiotemporal dynamic response to intensive land use in rural China. J. Environ. Manag..

[30] United Nations Environment Programme (1998). International Declaration on Cleaner Production.

[31] Shestalova V. (2003). Sequential malmquist indices of productivity growth: An application to OECD industrial activities. J. Prod. Anal..

[32] Elhorst J.P. (2012). Matlab software for spatial panels. Int. Reg. Sci. Rev..

[33] Lee G. (2006). The effectiveness of international knowledge spillover channels. Eur. Econ. Rev..

[34] Hsieh C., Klenow P.J. (2009). Misallocation and manufacturing TFP in China and India. Q. J. Econ..

[35] Wang X., Shao S., Li L. (2019). Agricultural inputs, urbanization, and urban-rural income disparity: Evidence from China. China Econ. Rev..

[36] Wu Y., Xi X., Tang X., Luo D., Gu B., Lam S.K., Vitousek P.M., Chen D. (2018). Policy distortions, farm size, and the overuse of agricultural chemicals in China. Prod. Natl Acad. Sci. USA.

[37] Hu J., Wang Z., Huang Q. (2021). Factor allocation structure and green-biased technological progress in Chinese agriculture. Econ. Res.-Ekon. Istraživanja.

[38] Chen M., Chen J., Du P. (2006). An inventory analysis of rural pollution loads in China. Water Sci. Technol..

[39] Hu J., Wang Z., Huang Q., Hu M. (2021). Agricultural trade shocks and carbon leakage: Evidence from China’s trade shocks to the Belt & Road economies. Environ. Impact Assess..

[40] Hu J., Pan X., Huang Q. (2020). Quantity or quality? The impacts of environmental regulation on firms’ innovation–Quasi-natural experiment based on China’s carbon emissions trading pilot. Technol. Forecast. Soc..

[41] Im K.S., Pesaran M.H., Shin Y. (2003). Testing for unit roots in heterogeneous panels. J. Econom..

[42] Choi I. (2001). Unit root tests for panel data. J. Int. Money Financ..

[43] Kao C. (1999). Spurious regression and residual-based tests for cointegration in panel data. J. Econom..

[44] Pedroni P. (2000). Critical values for cointegration tests in heterogeneous cointegrated panels with multiple regressors. Adv. Econom..

[45] Westerlund J. (2005). New simple tests for panel cointegration. Econom. Rev..

[46] You W., Lv Z. (2018). Spillover effects of economic globalization on CO_2_ emissions: A spatial panel approach. Energy Econ..

[47] Anselin L., Florax R.J.G.M. (1995). Small sample properties of tests for spatial dependence in regression models: Some further results. New Directions in Spatial Econometrics.

[48] Li X., Xu Y., Yao X. (2021). Effects of industrial agglomeration on haze pollution: A Chinese city-level study. Energy Policy.

[49] Li N., Jiang Y., Yu Z., Shang L. (2017). Analysis of agriculture total factor energy efficiency in China based on DEA and malmquist indices. Energy Procedia.

[50] Brümmer B., Glauben T., Lu W. (2006). Policy reform and productivity change in Chinese agriculture: A distance function approach. J. Dev. Econ..

[51] Savcı S. (2012). An agricultural pollutant: Chemical fertilizer. Int. J. Environ. Sci. Dev..

[52] Xiang T., Malik T.H., Nielsen K. (2020). The impact of population pressure on global fertiliser use intensity, 1970–2011: An analysis of policy-induced mediation. Technol. Forecast. Soc..

[53] Tang K., Gong C., Wang D. (2016). Reduction potential, shadow prices, and pollution costs of agricultural pollutants in China. Sci. Total Environ..

