# Supplementary material for: Spatial Spillover Effects of Resource Misallocation on the Green Total Factor Productivity in Chinese Agriculture
Source: Int J Environ Res Public Health. 2022 Nov 25;19(23):15718. doi: 10.3390/ijerph192315718 (PMC9736431; doi:10.3390/ijerph192315718)
Supplement: Supplementary file 1 [file ijerph-19-15718-s001.zip › ijerph-2007260-supplementary.pdf]

Table S1. Fertilizer loss rate in different regions.

Code	Region	Loss rate	
		TN	TP
I	Jiangsu, Beijing	0.3	0.07
II	Tianjin, Guangdong, Zhejiang, Shanghai	0.3	0.04
III	Hubei, Fujian, Shandong	0.2	0.07
IV	Hebei, Shaanxi, Liaoning, Yunnan, Ningxia, Hunan, Jilin, Inner Mongolia, Guizhou	0.2	0.04
V	Henan, Heilongjiang	0.1	0.07
VI	Anhui Hainan, Xinjiang, Shanxi, Guangxi, Gansu, Sichuan, Jiangxi, Chongqing, Qinghai, Xizang	0.1	0.04

Note: TN deontes total nitrogen, TP denotes total phosphorus.

Table S2. Pollution production coefficient of main crops (10^{-5}t/t).

Type	COD	TN	TP
Rice	3.1674	3.3872	0.0407
Wheat	4.0807	2.6523	0.1854
Corn	12.6158	11.4233	1.3097
Vegetable	7.6500	0.2484	0.1350
Oil crops	42.3043	206.3703	2.1438
Soybean	31.0165	49.4173	1.1491
Potato	0.5101	0.3349	0.1022

Note: COD denotes chemical oxygen demand, TN deontes total nitrogen, TP denotes total phosphorus.

Table S3. Nutrient loss rate of agricultural waste in different provinces (%).

Province	COD	TN	TP
Beijing	3.78	3.6	3.89
Tianjin	3.78	3.6	3.89
Hebei	16.41	14.05	10.13
Liaoning	8.27	6.72	3.75
Shanghai	11.21	8.82	4.79
Jiangsu	10.38	8.79	6.32
Zhejiang	7.83	6.64	4.37
Fujian	9.25	7.48	5.05
Shandong	11.57	10.39	8.61
Guangdong	10.18	8.11	4.73
Shanxi	13.24	10.46	5.24
Jiangxi	14.02	10.77	4.46
Jilin	8.27	6.72	3.75
Anhui	7.51	6	4.43
Heilongjiang	9.12	7.37	4.15
Henan	9.97	8.24	5.3
Hubei	8.05	6.15	2.35
Hunan	15.2	11.65	4.77
Sichuan	4.66	3.95	2.83
Inner Mongolia	0	0	0
Guangxi	10.18	8.11	4.73
Guizhou	8	7.25	8.75
Yunnan	3.78	3.6	3.89

Shaanxi	11.75	10.15	7.59
Gansu	9.06	7.72	5.21
Qinghai	0	0	0
Ningxia	3.15	2.8	2.85
Xinjiang	0	0	0
Hainan	10.1	8.2	6.9
Chongqing	4.66	3.95	2.83
Xizang	4.66	3.95	2.83

Note: COD denotes chemical oxygen demand, TN deontes total nitrogen, TP denotes total phosphorus.

Table S4. Variables and definition

Variable symbol	Variable name	Variable definition
Y	Expected output	Total agricultural output is deflated by the 1978 price index of primary industry output
TN	Unexpected output	Total nitrogen
TP	Unexpected output	Total phosphorus
COD	Unexpected output	Chemical oxygen demand
L	Land	The total sown area of crops
N	Labor	The number of people employed in agriculture
M	Machinery	The total power of agricultural machinery
F	Fertilizer	The discounted amount of fertilizer
GTFP	Green total factor productivity	Calculated by sequential DEA
Mis_L	Land misallocation	Calculated by Equation (17)
Mis_N	Labor misallocation	Calculated by Equation (17)
Mis_M	Machinery misallocation	Calculated by Equation (17)
Mis_F	Fertilizer misallocation	Calculated by Equation (17)
RPP	Rural population proportion	The ratio of the rural population to the total population
PGDP	Per capita GDP	The ratio of GDP to the total population
PD	Population density	The number of people per square kilometre
ER	Environmental regulation	The number of environmental protection establishments at the end of the year
Patent	Technological innovation	The number of domestic patent applications
FDI	Foreign direct investment	The foreign direct investment (USD 10000)

Table S5. Descriptive statistics of variables.

Variable	Unit	N	Mean	St. Dev.	Min	Max
Y	100 million yuan	6732	18.515	16.593	0.47	75.482
TN	t	6732	15812.95	20327.9	56.741	113611.8
TP	t	6732	937.176	1424.764	5.04	8462.468
COD	t	6732	21.846	25.704	0.077	127.171
L	Hectare	6732	375173.5	304534.2	6460	1351317
N	10 thousand people	6732	46.924	40.871	0.942	213.344
M	KW/h	6732	1896771	1852856	55238	9888656
F	t	6732	153317.5	175508.6	1103	1002894
GTFP	-	6732	0.702	0.251	0.239	1.649
Mis_L	-	6732	0.655	0.708	0.008	0.438
Mis_N	-	6732	0.656	0.798	0.009	0.443

Mis_M	-	6732	0.655	0.738	0.011	0.435
Mis_F	-	6732	0.747	0.897	0.01	0.491
RPP	-	6732	60.453	20.258	8.002	90.53
PGDP	10 thousand yuan/people	6732	0.553	0.577	0.027	4.677
PD	People/km^2	6732	339.405	276.827	0.001	1161.109
ER	Piece	6732	465.968	230.57	52	1145
Patent	Piece	6732	37452.52	81614.46	97	504500
FDI	100 million yuan	6732	1309.323	2072.411	12.732	9461.904
